# Tumor cell stemness in gastrointestinal cancer: regulation and targeted therapy

**DOI:** 10.3389/fmolb.2023.1297611

**Published:** 2024-02-22

**Authors:** Kangqi Yang, Tuo Yi

**Affiliations:** ^1^ School of Basic Medical Sciences, Fudan University, Shanghai, China; ^2^ Department of General Surgery, Zhongshan Hospital, Fudan University, Shanghai, China

**Keywords:** gastrointestinal tumor, cancer stem cell, tumor heterogeneity, tumor microenvironment, epithelial-to-mesenchymal transition, cancer-associated fibroblast

## Abstract

The cancer stem cells are a rare group of self-renewable cancer cells capable of the initiation, progression, metastasis and recurrence of tumors, and also a key contributor to the therapeutic resistance. Thus, understanding the molecular mechanism of tumor stemness regulation, especially in the gastrointestinal (GI) cancers, is of great importance for targeting CSC and designing novel therapeutic strategies. This review aims to elucidate current advancements in the understanding of CSC regulation, including CSC biomarkers, signaling pathways, and non-coding RNAs. We will also provide a comprehensive view on how the tumor microenvironment (TME) display an overall tumor-promoting effect, including the recruitment and impact of cancer-associated fibroblasts (CAFs), the establishment of an immunosuppressive milieu, and the induction of angiogenesis and hypoxia. Lastly, this review consolidates mainstream novel therapeutic interventions targeting CSC stemness regulation.

## 1 Introduction

Gastrointestinal (GI) cancer refers to the malignant tumors in the GI tract and its accessory organs, including the esophagus, stomach, small intestine, colon, rectum, anus, liver, gall bladder and pancreas. Cancer statistics issued by American Cancer Society (ACS) estimated 348,840 new cases and 172,010 deaths cause by GI cancer in the United States in 2023 (Siegel et al., 2023). The incidence of GI cancer in the United States between 2015 and 2019 was as high as 50.9 per 100,000, and the mortality rate from 2016 to 2020 was 22.5 per 100,000. Such high morbidity and mortality urges the need to understand the molecular mechanisms underlying the initiation, migration and therapeutic resistance of GI cancer.

Surgical resection presently stands as the standard therapeutic modality for early-stage GI cancer. However, in cases where complete resection is unattainable, the therapeutic focus pivots toward employing adjuvant therapies, particularly chemotherapy and immunotherapy, to extend patient survival. Despite myriad clinical endeavors targeting cancer, sustainable outcomes remain elusive due to the emergence of therapy resistance in cancer cells. With an attempt to uncover the underlying mechanisms, multiple researches have focused on tumor heterogeneity, a salient hallmark of malignancy. Tumor heterogeneity manifests at both intertumoral and intratumoral levels. The former denotes individual variations, while the latter exhibits both as spatial heterogeneity, which refers to a scattered distribution of genetically distinctive cell subpopulation across or within a tumor site, and temporal heterogeneity, described as the temporal variation in the genetic alterations in one individual tumor (Dagogo-Jack and Shaw, 2018). Focusing on the dynamics in cancer development, the cancer stem cell (CSC) model has gained widespread acceptance. According to this paradigm, CSCs, resembling normal stem cells, are a group of cells characterized by an uncontrolled proliferation potential and an indefinite capacity to drive the growth of new tumors (Shibue and Weinberg, 2017).

Fueled by the notion that tumors are essentially aberrant organs driven by tumor-initiating cells, researches begin to aim for analogies between normal stem cells and tumorigenic cells (Reya et al., 2001). The CSC theory, initially elucidated in hematopoietic malignancy (Lapidot et al., 1994), gains further credence through the evidence from the hierarchical arrangement of phenotypically varied cancer cells (Prasetyanti and Medema, 2017). Termed also as tumor initiating cells, CSC has been shown by mounting evidence to exhibit high tumorigenicity (Huang et al., 2020; Walcher et al., 2020). Extensions of this concept further proposed CSC’s role in therapy resistance, ascribing the limited efficacy of conventional therapies to their impotency in targeting CSC (Dean et al., 2005; Shibue and Weinberg, 2017; Dagogo-Jack and Shaw, 2018). Evidence revealing CSC’s high resistance in its dormant state (Aguirre-Ghiso, 2007; Talukdar et al., 2019), and the notion that reactivated CSCs continue to act as tumor precursors (Shibue and Weinberg, 2017), prompted extensive investigations on its significance as a trigger of cancer relapse. In summary, CSC stemness is defined as its function in tumor initiation, metastasis, therapeutic resistance and reoccurrence, making it one of the most significant subjects for understanding tumorigenesis.

Of all biological programs regulating CSC stemness, the most attributable is epithelial-to-mesenchymal transition (EMT), a process critical for embryonic development, wound healing and tissue regeneration (Shibue and Weinberg, 2017). As the name suggests, the program drives epithelial cells to undergo a reduction in apicobasal polarity and intracellular adhesion (Yilmaz and Christofori, 2009), consequently offering them mesenchymal-like properties including higher invasiveness and motility (Shibue and Weinberg, 2017). Aberrantly triggered EMT has been elucidated to have significant function in CSC stemness activation, inducing cell renewal and therapeutic resistance (Wilson et al., 2020; Zhang et al., 2021). Via signaling pathways, EMT transcription factor ZEB1, TWIST and SNAIL undergoes alterations (De Craene and Berx, 2013), which will suppress epithelial-associated proteins such as E-cadherin, and in turn upregulate genes encoding mesenchymal-associated proteins, including vimentin and N-cadherin (De Craene and Berx, 2013).

**FIGURE 1:**
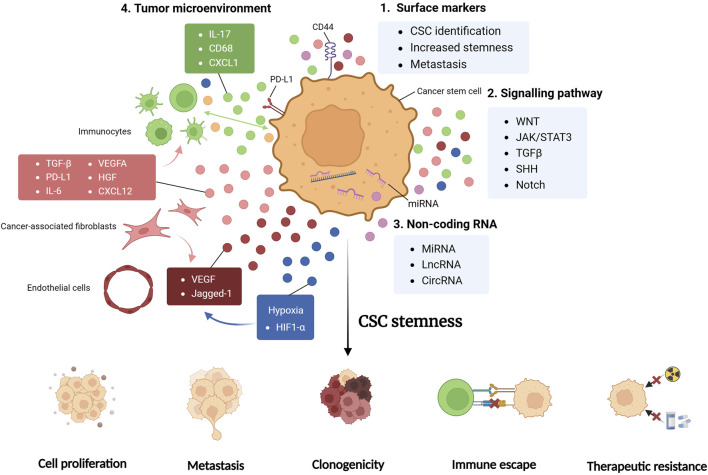
CSC regulators. Cancer stem cell (CSC) properties are regulated by multiple factors, with the integrative effective of promoted stemness, exhibited as enhanced cell proliferation, tumor metastasis, clonogenicity, immune evasion and therapy resistance. CSC surface markers, including CD44, are critical for the stemness maintenance and metastasis enhancement of CSC, and can serve as means of CSC identification in researches (1). Signaling pathways are formed by cytokines and chemokines secreted from tumor cells and various cells in tumor microenvironment (TME). These substances are involved in complex crosstalk and cellular interactions, shaping the CSC niche significantly (2). Non-coding RNA (ncRNA) including miRNA, lncRNA and circRNA are found to exert both pro-tumorigenic and antitumoral effects, fine-tuning TME and CSC properties. Some ncRNAs directly modulates genetic alterations, while others function by paracrine signaling (3). TME harbors various cell types, each secreting multiple factors and engages in complex crosstalk regulating CSC stemness (4). *JAK/STAT3, Janus kinase/signal transducer and activator of transcription 3; TGFβ, transforming growth factor beta; SHH, sonic hedgehog; miRNA, micro RNA; lncRNA, long non-coding RNA; circRNA, circular RNA; CXCL, CXC-chemokine ligand; PD-L1, programmed cell death 1 ligand 1; VEGFA, vascular endothelial growth factor A; HGF, hepatocyte growth factor; HIF1-α, hypoxia-inducible factor-1α.*

The CSC model has become a mainstream concept featured in paramount researches ever since its first release. However, our knowledge about the underlying molecular mechanism of CSC regulation is still limited, due to the lack of connections built between the potential regulators. Meanwhile, based on the spatial heterogeneity of CSC, it is also critical to study the diverse regulatory mechanisms regarding tissue specificity. Thus, a comprehensive analysis of CSC regulators is needed to conclude the complex crosstalk and provide clear insights for therapeutic strategies. In this review, we summarize the current mainstream findings of factors engaged in the regulation of CSC properties in GI tumors, including its biomarkers [Figure 1(1)], signaling pathways [Figure 1(2)] and non-coding RNAs (ncRNAs) [Figure 1(3)]. We will also closely investigate the niche CSCs reside in to assess the complex crosstalk between multiple regulators [Figure 1(4)]. By featuring both organ specificity and cross-tissue comparison, we will provide insights in the plasticity and mutual properties exhibited by CSCs in different GI organs, and propose clues for therapies exclusively targeting GI tumors.

## 2 CSC surface markers

Identification of CSC, which currently relies heavily on cell surface markers, is crucial for CSC isolation and early detection. Recent literature has selected a list of biomarker candidates highly correlated to elevated CSC phenotypes and worsened prognostic results (Dalerba et al., 2007; Chu et al., 2009; Sun et al., 2013).

### 2.1 CD44

CD44, one of the most widely identified CSC markers, is a transmembrane glycoprotein originally expressed on epithelial cell surface for regulating intracellular interactions and immunocyte activation (Hassn Mesrati et al., 2021). Extensive literature reports have highlighted an upregulation of CD44 mRNA expression across multiple cancer types, particularly on cells displaying EMT phenotype.

The structural and functional features of CD44 render it particularly well-suited for facilitating CSC metastasis. As a hyaluronic acid receptor, CD44 engages with multiple ligands, adeptly mediating intracellular interactions that activate cancer cell migration and metastasis (Yu and Stamenkovic, 1999). Meanwhile, CD44 participates in metabolic regulation, inducing chemotherapy resistance by suppressing intracellular reactive oxygen species (ROS) in cancer cells (Tamada et al., 2012).

By interacting with downstream pathways, CD44 has been found to influence stemness and migration in various tumor cell types. In a study isolating CD44^+^ cells from human gastric cancer (GC) cells (Sun et al., 2013), Sun et al. conducted a spheroid colony formation assay, revealing heightened stemness and elevated invasiveness *in vitro* and *in vivo*. Concordantly, CD44 knockdown resulted in diminished stemness and migration of GC tumor cells, confirming the necessity of CD44 in CSC tumorigenesis (Takaishi et al., 2009). Research comparing colorectal cancer (CRC) xenograft derived from different percentage of EpCAM/CD44 cells discovered that EpCAM^high^/CD44+ cell group possesses the highest level of tumorigenicity (Dalerba et al., 2007). Additionally, CRC cell fluorescence-activated cell sorting (FACS) analysis identified the CD44^+^ cell subgroup as possessing high clonogenicity and *in vivo* tumorigenicity (Chu et al., 2009). In hepatocellular carcinoma (HCC), CD44 also induces a mesenchymal phenotype by activating TGFβ pathway, consequently promoting cancer migration and metastasis (Mima et al., 2012). Subsequent analysis of HCC patient samples revealed high CD44 expression as a poor prognosis indicator, confirming its clinicopathological significance. Furthermore, the separation of pancreatic ductal adenocarcinoma (PDAC) cell lines with different CD44 level uncovered a correlation between high CD44 level and enhanced CSC-like properties, including elevated tumorigenicity, clonogenic abilities, and therapy resistance *in vivo* (Zhao et al., 2016). Previous investigations into CD44-expressing cells in PDAC also unveiled its greater EMT phenotype and invasiveness *in vitro* (Mani et al., 2008).

Notably, CD44 isoforms exhibit both distributional and functional heterogeneity. CD44 has two major isoforms, CD44 standard (CD44s) and CD44 variants (CD44v). While CD44v exhibit significantly greater tumorigenicity than CD44s in pancreatic carcinoma (Li et al., 2014), the roles are reversed in HCC and gall bladder carcinoma (GBC), where CD44s acts as the predominant CSC-promoter (Mima et al., 2012; Miwa et al., 2017). Due to the heterogeneity of CD44 isoforms, closer investigations are needed before therapeutically targeting CD44 to ensure the treatment’s targeting accuracy and organ specificity.

### 2.2 CD133

CD133 is one of the most well-studied CSC markers. This cell surface marker has been implicated in instigating an upregulation of tumorigenic factors in GC, including EMT-associated SNAIL and N-cadherin, along with the tumor development promoters Oct-4 and c-myc (Soleimani et al., 2022). Similarly, CD133 can mediate tumor stemness and metastatic properties in CRC by exosome secretion (Zhao et al., 2020). Clinically, meta-analysis on GC patients also observed increased lymphatic metastasis (Yiming et al., 2015) and worsened 5-year survival rate among the patients harboring CD133-positive cells (Wen et al., 2013). Similar clinicopathological correlation was recorded in CRC, PDAC and EC patients (Chen et al., 2013b; Wen et al., 2013; Wang et al., 2019b), suggesting the high prognostic value of CD133.

CD133 has been demonstrated to promote the proliferation and CSC-like traits across diverse tumor types, which positions CD133 as a key candidate for tumor identification and therapeutic targeting. However, CD133 has been categorized by some as a less effective marker, as several studies fail to observe a distinguishable tumorigenicity increase in CD133+ cells compared to CD133-groups (Dalerba et al., 2007), possibly due to distributional heterogeneity.

### 2.3 EpCAM

The epithelial cell adhesion molecule (EpCAM) is found to be expressed on CSC across various cancer types. Based on the notion that EpCAM serves as a marker of normal hepatic stem cells, researchers sought the mechanism of tumorigenicity of EpCAM in the pre-cancerous stage of advanced cirrhosis. Investigations into EpCAM + cells from advanced cirrhosis revealed an elevated expression of the Notch gene, accompanied by enhanced self-renewal *in-vitro* and an increased resemblance to HCC regarding genetic products (Khosla et al., 2017). Moreover, Yamashita et al. recorded an increased invasiveness of HCC EpCAM cells in NOD/SCID mouse model, elucidating the regulatory mechanism through the Wnt/β-catenin signaling pathway (Yamashita et al., 2009). EpCAM is also among the first markers to effectively identify CSCs in PDAC (Ishiwata et al., 2018), CRC (Dalerba et al., 2007) and GC (Dai et al., 2017), making it a promising candidate for the identification of CSCs in GI cancers.

### 2.4 ALDH

Aldehyde dehydrogenase (ALDH), an enzyme integral to diverse metabolic processes (Toledo-Guzmán et al., 2019), has only recently gained recognition for its involvement in CSC identification, as cumulative evidence suggesting it as a major regulator governing tumorigenic properties (Mele et al., 2018; Toledo-Guzmán et al., 2019). In GC, ALDH^high^ cells were observed to have greater sphere-forming ability *in vitro* and elevated tumorigenicity *in vivo*. The orchestrated regulation of ALDH aligns with the activities of Notch and Sonic Hedgehog (SHH) signaling pathways, emphasizing its critical role in mediating downstream cellular activities (Nishikawa et al., 2013). Furthermore, ALDH has emerged as a promising marker for identification in esophageal cancer (EC) (Islam et al., 2015) and PDAC (Rasheed et al., 2010).

Interestingly, even as the majority of studies concluded ALDH as a key contributor to tumorigenesis and poor clinical outcomes, a few studies posit alternative perspectives. An analysis of 1420 CRC tissue microarrays by Lugli et al. failed to establish a significant correlation between ALDH expression and patient survival rate (Lugli et al., 2010). Meanwhile, a comprehensive analysis of cell surface markers in PDAC documented the ALDH expression abundance in normal pancreatic tissue as similar to that of the cancer tissue, challenging its exclusivity as a CSC marker in PDAC (Ishiwata et al., 2018). Although these reports do not diminish the potential of ALDH as a CSC marker, they underscore the importance of developing a universal CSC identification strategy, and indicate the challenges of CSC identification posed by its spatial heterogeneity.

### 2.5 Transcriptional factors

Despite the aforementioned surface markers, CSC transcriptional factors are also valuable for CSC identification and therapeutic targeting. Sox2, a member of the SRY-related HMG-box (SOX) family, is among the most extensively discussed factors. Employing an *in-vitro* cell culture model, Lundburg et al. substantiated that Sox2-expressing cells exhibit CSC-like features, which is concomitant with a reduced level of CDX2, a state typically observed in late-stage CRC patients (Lugli et al., 2010). These findings are in line with the research conducted by Takeda et al., which also elucidated Sox2’s role in the maintenance of stemness and the regulation of differentiation states (Takeda et al., 2018). Similarly, in GC and PDAC, expression of Sox2 correlates with enhanced self-renewal ability, metastatic potential and therapy resistance (Wuebben et al., 2016; Zhang et al., 2016; Sharma et al., 2019).

While the landscape of cell marker research continues to expand, biomarkers are not completely reliable for CSC identification. As previously highlighted, the substantial heterogeneity among tumor subtypes and the phenotypic plasticity inherent in CSCs can compromise the efficacy of surface markers. Due to CSC plasticity, the ablation of biomarkers may not completely diminish the CSC-like traits. A study by Huang et al., in 2013 challenged the effectiveness of biomarkers in distinguishing CSCs. They examined the tumorigenic properties of CSC+ (narrowly defined as tumor cells positive of the well-studied CSC markers) and CSC- cells, and found no significant pathological difference after co-transplanting the 2 cell groups in mouse models (Huang et al., 2013). Conclusions from this research is limited, given its inclusion of only a few markers as “CSC+” standards, while numerous novel biomarkers have been discovered and investigated since its publication. Nonetheless, the findings prompt careful considerations when utilizing surface markers for CSC identification or developing corresponding therapies, regarding the high plasticity and heterogeneity associated with CSCs.

## 3 Signaling pathways

Multiple pathways have been demonstrated to play critical roles in promoting tumorigenic properties, including the elevation of CSC biomarker expression, the regulation of CSC activity, the augmentation of EMT process and the establishment of an immunosuppressive TME. Moreover, compared to cell biomarkers, signaling pathway components are less influenced by subtype heterogeneity and CSC plasticity, therefore possess higher stability in tumor identification and therapeutic targeting.

### 3.1 WNT

WNT, a highly complex pathway involved in various biological processes, is crucial for determining cell fate and inducing CSC properties. Aberrant activation of WNT pathway has been reported across multiple cancer types, including CRC (Pan et al., 2017), EC (Chu et al., 2022) and GC (Katoh and Katoh, 2022).

WNT is critical for the proliferation/dormancy state transformation of CSC. This process is of great significance to CSC stemness maintenance and therapeutic resistance, as proliferative CSCs are highly vulnerable to therapies compared to their dormant state (Phan and Croucher, 2020). This transformation is mediated by a dynamic interplay between the two states of WNT, namely, the canonical and noncanonical signaling pathway (Katoh, 2017). The former can directly elevate the transcription level of oncogene MYC and cell cycle activator CCND1 (Katoh and Katoh, 2007), meanwhile activating β-catenin to enhance CSC expansion (Katoh and Katoh, 2017). The noncanonical pathway, on the other hand, contributes to β-catenin degradation and CSC dormancy (Katoh and Katoh, 2017).

Multiple CSC biomarkers rely on WNT pathway to regulate their expression and function. Ishimoto et al. has demonstrated on GC-imitating K19-Wnt1/C2mE mouse model that WNT signaling upregulates CD44^+^ cell residing at gastric gland squamo-columnar junction (SCJ), consequently activating the quiescent tissue to adopt a malignant growth pattern (Ishimoto et al., 2010). Similar regulatory effects have been observed in CRC and PDAC, where WNT promotes tumorigenesis by enhancing CD44 activities (van de Wetering et al., 2002; Wang et al., 2015). WNT can also regulate EpCAM expression and activity in HCC, consequently affecting the stem cell-like features of the EpCAM + subgroup (Yamashita et al., 2009).

Beyond its influence on biomarkers, WNT regulates multiple immune-relevant factors to mediate tumor immune evasion. Canonical WNT signaling pathway is shown to directly increase PD-L1 expression (Katoh and Katoh, 2022) and Treg-recruiting C-C motif chemokine ligand (CCL) 28 in GC (Ji et al., 2020). This pathway also facilitates Treg production by limiting IL-12 secretion (Spranger et al., 2017) and downregulating dendritic cell (DC)-recruiting chemokines CCL4 and CCL5 (Katoh and Katoh, 2022). Interestingly, previous researches reported that WNT exhibit a positive effect on T cell maturation and activation (Gattinoni et al., 2010), and that WNT downregulates FOXP3 to inhibit Treg immunosuppression (Zhou et al., 2022b). These findings suggest that WNT can display immune-supportive functions under certain conditions. Collective insights into WNT plasticity are needed to underscore the potential for spatiotemporal regulation of WNT signaling to improve tumor immunotherapy.

### 3.2 JAK/STAT

The Janus kinase/signal transducer and activator of transcription 3 (JAK/STAT3) pathway is crucial in tumorigenesis, exerting regulatory control over tumor initiation, progression and immune responses. Initiated by IL-6, activation of the JAK/STAT axis begins from an induced conformational change of IL-6 receptor, which then activates JAK and initiates the latent transcription factor STAT3 activity (Jones and Jenkins, 2018). The IL-6 family, encompassing IL-11, IL-27, and IL-31, emerges as a set of key regulators influencing immune responses, pathway signaling and CSC expansion (Jones and Jenkins, 2018; Jin, 2020).

By upregulating CD44 ligands and forming a CD44/acetylated STAT3 complex, the IL-6/STAT3 pathway promotes biomarker expression and enhances CSC survival (Lee et al., 2008; Su et al., 2011). Additionally, STAT3 is among the pivotal transcription factors in EMT induction. A study on mouse liver has demonstrated that STAT3 upregulation, triggered by Twist1 expression, activates tumorigenesis by inducing EMT (Uthaya Kumar et al., 2016). Moreover, the JAK/STAT3 pathway mediates therapeutic resistance. As demonstrated by Ma et al., JAK/STAT3-activating IL-11 promotes acquired chemoresistance in GC, followed by a cascading effect that consequently induces anti-apoptosis and diminishes therapeutic outcome (Ma et al., 2019a).

Actively engaged in immunocyte crosstalk, the JAK/STAT3 pathway plays an important role in shaping immunosuppression and indirectly inducing CSC expansion. Members of the IL-6 family, primarily produced under inflammatory states (Tanaka et al., 2016; Yoshida, 2020), contribute to promoting EC and CRC immunosuppression by blocking DC maturation and inducing the M2 polarization of tumor-associated macrophage (TAM) (Wang et al., 2014; Higashino et al., 2019). IL-6 also upregulates cell programmed death 1 (PD-1) expression on CD8 T cells and cell programmed death 1 ligand 1 (PD-L1) on tumor cells, which leads to cytotoxic cell dysfunctionality (Ju et al., 2020). Additionally, the IL-6/STAT3 axis mediates TAM’s CSC-promoting property. A coculture of CD44^+^ human HCC cells and TAMs has shown positive correlations between an elevated IL-6 level and the expansion of the CD44^+^ cell group, implicating STAT3’s critical role in mediating TAM-CSC crosstalk (Wan et al., 2014).

### 3.3 TGFβ

Transforming growth factor beta (TGFβ) is a cytokine widely expressed in normal tissues, serving key modulatory functions of cell growth, immune responses and intracellular communications (Kubiczkova et al., 2012). In tumor tissues, aberrantly-triggered TGFβ is also engaged in activities of many tumor-associated cells including cancer-associated fibroblasts (CAF) and TAM (Trelford et al., 2022).

TGFβ release is stimulated by extracellular matrix (ECM) degradation. When ECM degradation reaches a certain rate, CAFs are triggered to release TGFβ, inducing the production of pro-oxidant enzymes and ROS, while upregulating the fibrogenesis factors. These effects culminate in desmoplastic aggregation and ECM stiffness promotion (Fuyuhiro et al., 2011; Mortezaee, 2021), which induces the development of CSC-like traits.

TGFβ orchestrates the initiation of EMT across various GI malignancies, including GC, CRC and HCC (Calon et al., 2015; Krzysiek-Maczka et al., 2019; Jeng et al., 2023). It directly upregulates the production of multiple EMT transcription factors such as SNAIL, TWIST, SLUG and ZEB1 (Lee and Massague, 2022; Trelford et al., 2022), and also promotes EMT by introducing epigenetic alterations. Via the SMAD signaling pathway, TGFβ downregulates DNA demethylation, inducing a hypermethylated state of multiple promoters of genes encoding EMT factors. For example, by inhibiting DNA methyltransferases (DNMT)-induced DNA methylation, TGFβ upregulates CD133 expression (You et al., 2010), enhancing HCC-cell metastasis. Moreover, Malfettone et al. reported a stimulatory effect of TGFβ-induced EMT on CD44 expression in HCC, indicating TGFβ′s role in upregulating CSC biomarkers (Malfettone et al., 2017). Tumor cells undergoing TGFβ-induced EMT exhibit increased resistance to therapies including chemotherapy and radiation, establishing a correlation between high TGFβ levels and unfavorable prognosis (Zheng et al., 2015).

TGFβ also plays a critical role in shaping the tumor immune landscape and mediating CSC immune escape. Targeting cytotoxic T cells, TGFβ can directly diminish their aggressiveness by hindering their responses and inducing apoptosis (Busenhart et al., 2022; Chen et al., 2022). Meanwhile, TGFβ can interfere with DC activities, inhibiting its proliferation, antigen presenting function and overall mobilization (Esebanmen and Langridge, 2017; Peng et al., 2022), thereby obstructing the differentiation process of adaptive immune cells. Similarly, TGFβ dampens natural kill (NK) cell stimulation and interferon-gamma (IFNγ) production (Hayashi et al., 2003; Peng et al., 2022), attenuating NK cell effects in cytolysis and lymphocyte mediation (Castro et al., 2018). Furthermore, TGFβ generates immunodeficiency by inducing cell differentiation in an immunosuppressive direction. For instance, TGFβ-recruited tumor-associated neutrophils (TANs) generally exhibit decreased cytotoxicity, indicating their differentiation in a tumorigenic fashion (Fridlender et al., 2009; Song et al., 2021).

Notably, a number of TGFβ-mediated activities result in a positive feedback loop. For example, while TGFβ interacts with DC to inhibit its normal immune functions, it also upregulates the DC-derived TGFβ level, enhancing its effects in DC regulation (Esebanmen and Langridge, 2017). The same mechanism can be observed in TAN and CAF (Esebanmen and Langridge, 2017; Peng et al., 2022), where TGFβ interactions can trigger autocrine TGFβ loops, magnifying the tumorigenic effects.

### 3.4 Sonic hedgehog

Throughout mammalian embryogenesis, the SHH signaling pathway emerges as a key mediator orchestrating tissue differentiation and human foregut formation. Consequently, an abundant level of SHH can be found in human gastric mucosa, contributing to epithelial growth, gastric acid production and potential neoplastic transformation (Merchant, 2012; van den Brink et al., 2002). Additionally, SHH-mediated organogenesis is also observed in the maturation of the pancreas, development of the gallbladder, and the repairing of liver tissue (Swiderska-Syn et al., 2016; Jeng et al., 2020).

While SHH activity typically remains dormant in mature adults, aberrations in the SHH pathway can reawaken its influence, promoting cancer initiation and progression (Jeng et al., 2020). For instance, mutated SHH pathways have been discovered in the activation of human pancreatic myofibroblast invasion *in vitro*, underscoring SHH’s role in fostering desmoplasia in PDAC and subsequently supporting CSC metastasis (Bailey et al., 2008). Additionally, research by Jeng et al. has demonstrated the expansion of HCC *in vivo* following SHH gene alterations, suggesting its regulatory impact on HCC growth (Jeng et al., 2015). Moreover, by employing xenotransplantation in patient-derived CRC organoids, Regan et al. has elucidated SHH’s role in promoting CSC survival via crosstalk with the WNT pathway (Regan et al., 2017).

### 3.5 Notch

The Notch signaling pathway regulates genes associated with stem cell maintenance, exerting critical influence on cell proliferation, differentiation and tissue maturation (Zhou et al., 2022a). Upon initiation from receptor-ligand binding event, the Notch pathway sets off a series of downstream cascading effects. The ultimate product translocates to the nucleus (Andersson et al., 2011; Zhou et al., 2022a), engaging in transcriptional interactions that lead to the upregulation of genes such as HES, MYC, and CDKN1A, which encode proteins essential for maintaining CSC phenotype, initiating oncogenesis, and inhibiting apoptosis (Clara et al., 2020).

Notably, the Notch pathway exhibits dual effects in tumor progression, acting as an oncogene or a tumor suppressor depending on varied receptor/ligand expressions or upstream regulators (Li et al., 2023c). Aberrant overexpression of Notch, as observed in GC, PDAC, CRC and HCC (Vinson et al., 2016; Hibdon et al., 2019; Tyagi et al., 2020; Chung and Xu, 2023; Jeng et al., 2023), relates the Notch gene to tumor types characterized by increased aggressiveness, promoted drug resistance and poor clinical prognosis. The Notch pathway upregulates CSC activities by interacting with CSC surface markers. Based on an observed increase of Notch1 in ALDH^high^ cells in GC, Nishikawa et al. proposed that Notch pathway regulates CSC maintenance and induces chemotherapy resistance (Nishikawa et al., 2013). Abundant research on Notch-CSC interactions also reveals its contribution in promoting CSC self-renewal, tumor angiogenesis, and metastasis (Miao et al., 2017; Akil et al., 2021). Regarding immune system regulation, Notch is also found to induce immune evasion by promoting TAM activation and inhibiting T cell proliferation (Palaga et al., 2018; Meng et al., 2022).

However, Notch signaling also exhibits anti-tumor properties by influencing NK cell proliferation and DC cell maturation (Felices et al., 2014; Meng et al., 2016). Understanding the precise influence and regulators of Notch’s dual effects in tumors, particularly GI tumors, is still in its early stages. Further investigations are needed to elucidate the complex mechanisms underlying the Notch signaling pathway and to gain a comprehensive understanding of tumor heterogeneity.

Interplay among diverse pathways contributes to coordinated regulation of tumorigenesis. For instance, the IL-6/JAK/STAT3 pathway can induce a cascading effect, resulting in the upregulation of TGFβ and the collaborative orchestration of EMT in HCC (Wang et al., 2018a). Conversely, a TGFβ-induced CAF has been identified as a source of IL-11, which can activate STAT3 and further enhance metastasis in CRC (Calon et al., 2012). Therefore, a holistic perspective is imperative for the comprehensive exploration of the regulatory roles of signaling pathways in tumorigenesis.

## 4 Non-coding RNA

While more than 90% of genomes undergo a transcription process, only 2% are determined to be protein-encoding (Wilusz et al., 2009). With accumulating evidence revealing their cell-specific expression and distribution, the blank spaces left by the non-coding RNAs (ncRNAs) has recently drawn increasing attention. ncRNA includes microRNA (miRNA), long non-coding RNA (lncRNA) and circular RNA (circRNA), all proven to be critical in physiological and pathological processes.

### 4.1 MiRNA

MiRNAs, a group of small ncRNA with approximately 22 nucleotides (Bartel, 2004), block gene expression by binding to the 3′-UTR of target mRNA (Pan et al., 2021). Their impacts are highly heterogenous across different tumors, some promoting cancer stemness and drug resistance, while others demonstrating overall tumor suppressive effects.

MiRNAs modulate the expression of CSC biomarkers to exert control over cancer progression. For instance, miR-328 acts as an effective inhibitor of CD44 (Xue et al., 2016). Its reduced levels are associated with higher GC recurrence rates and enriched CD44^+^ cells, establishing a negative correlation between miR-328 and cancer development (Ishimoto et al., 2014). Targeting CD44, miR-145 also exerts tumor-suppressive effects, attenuating EMT responses and reducing chemotherapy resistance (Zeng et al., 2017; Zhu et al., 2018; Mozammel et al., 2023). Similar effects were observed in miR-17 and miR-1185, which inhibits the expression of SOX2 and CD24, respectively (Li et al., 2015a; Wang et al., 2020a).

Multiple pathway signaling reactions are also regulated by miRNA. MiR-1247-5p is found to be downregulated in HCC patients, suggesting its negative correlation with tumor progression. Further investigation unveiled its target as Wnt3, the inhibition of which induces CSC apoptosis *in vitro* and suppresses cancer cell growth *in vivo* (Chu et al., 2017). Similarly, miR-4319 emerges as a potent anticancer factor. Targeting multiple genes associated with GI tumors, it inhibits cell proliferation, reduces CSC stemness and suppresses EMT in HCC (Han et al., 2019), CRC (Huang et al., 2019a) and EC (Hu et al., 2020). Additionally, miR-30a and miR-9 downregulates TM4SF1 (Park et al., 2016; Park et al., 2017), a key oncogenic gene known for its role in activating the Wnt/β-catenin pathway and enhancing EMT (Tang et al., 2020). MiRNAs also exert indirect regulatory effects via responding to TME alterations. MiR-215 is upregulated by hypoxia, a tumor-initiating condition in TME. However, as miR-215 operates in a negative feedback loop, the elevation of which curtails CSC biomarker LGR5 and suppresses tumor initiation (Ullmann et al., 2019).

Though the majority of CSC-related miRNAs demonstrate tumor-suppressive effects (Pan et al., 2021), a few exhibit CSC-promoting functions. Aberrant miR-5188 expression promotes HCC stemness by mediating β-catenin translocation (Lin et al., 2019), while miR-577, a highly heterogenous factor, shows an upregulation in GC (Luo et al., 2019), reflecting the complex and heterogeneous nature of these regulatory molecules. Further investigations are needed to clarify the mechanisms underlying the dysfunctional expression of CSC-associated miRNAs and explore therapeutic strategies employing tumor suppressor miRNAs or inhibiting oncomiRNAs.

### 4.2 LncRNA

LncRNAs are noncoding transcripts that contain more than 200 nucleotides (McCabe and Rasmussen, 2021). By initiating chromatin modification on protein-coding genes, lncRNA can exert alterations in transcription, pro-transcription and translation (Castro-Oropeza et al., 2018; McCabe and Rasmussen, 2021).

LncRNAs play an active role in determining cellular fate and engaging directly with oncogenic regulatory factors. Among them, Hox transcript antisense intergenic RNA (HOTAIR) has been identified in CRC, GC, PDAC and HCC (Castro-Oropeza et al., 2018). It regulates EMT-associated gene expression by recruiting chromaffin-mediating enzymes and inducing the activation or silencing of target genes. Through transcriptional alterations, HOTAIR upregulates factors such as ZEB1, TWIST and SNAIL, meanwhile stimulates TGFβ1 pathway (Pádua Alves et al., 2013), instigating EMT and CSC clonogenicity, thereby promoting tumor metastasis and growth (Castro-Oropeza et al., 2018). Similarly, metastasis-associated lung adenocarcinoma transcript 1 (MALAT-1) was found to promote tumorigenesis in CSC derived from PDAC, CRC and GC (Hu et al., 2016; Chen et al., 2017; Wu et al., 2018). Same as most oncogenic lncRNAs, MALAT-1 elevates the expression of EMT-associated factors (McCabe and Rasmussen, 2021), maintains CSC renewal and induces angiogenesis by stabilizing SOX2 mRNA (Jiao et al., 2015).

Moreover, lncRNAs can function as miRNA sponges with specific effects. CAF-derived lncRNA H19 amplifies CRC stemness by acting as miRNA-41 sponge, whose enrichment activates the β-catenin pathway, elevating CSC stemness and chemoresistance *in vitro* and *in vivo* (Ren et al., 2018). Similarly, lncRNA NALT1 correlates with increased mortality rate in GC and CRC patients via sponging miRNA574-5p, which mediates the elevation of PEG10, a gene responsible for CSC proliferation and invasion (Ye et al., 2022).

### 4.3 CircRNA

CircRNAs are a class of single-stranded nucleotides characterized by their covalently closed loop structure. Each circRNA undergoes back-slicing, in which the absent 5′ and 3’ of pre-mRNA bind together to form a structure with enhanced stability (Liu and Chen, 2022).

With their abundant expression levels, circRNA exhibits tumor-promoting properties by functioning as regulators of transcription and splicing. CRC-derived circCTIC1 was found to correlate to worse prognosis of CRC patient. Research uncovered its interaction with a protein complex in the nucleus, directly triggering the expression of c-Myc, a significant tumor-promoting gene (Zhan et al., 2019). Another circRNA, circFNDC3B, has been associated with EMT levels in GC, contributing to the downregulation of E-cadherin expression and interacting with CD44 mRNA to bolster aggressive invasion (Hong et al., 2019). Another mechanism proposes that, the generation of circRNAs through the back-splicing mechanism disrupts alternative linear splicing of pre-mRNA, potentially influencing gene expression levels (Samavarchi Tehrani et al., 2023).

Similar to lncRNA, circRNA can also be employed as miRNA sponges. In a genome-wide sequencing study focused on CRC-associated RNAs, Rengganaten et al. elucidated a circRNA-miRNA-mRNA axis mainly mediated by circRNA hsa_circ_0066631 and hsa_circ_0082096. These circRNAs take effects via the downregulation of target miRNAs, thus inhibiting the degradation of mRNAs encoding CSC-promoting proteins, including activators of WNT/β-catenin and TGFβ (Rengganaten et al., 2020). Notably, though circRNA is mostly investigated as ncRNA, emerging evidence suggests their mRNA-resembling potential in protein encoding (Pamudurti et al., 2017), opening a promising field for further research.

## 5 Tumor microenvironment

Tumor microenvironment (TME) encompasses a diverse array of non-tumorigenic cells, including immune cells, endothelial cells, mesenchymal stroma-like cells (MSC) and CAF, all embedded in a matrix of structured proteins forming ECM (Osipov et al., 2019). TME form specialized compartments called niches, which reside CSCs and regulate cellular activities by intracellular contacts or secreted factors including cytokines, growth factors and hormones (Plaks et al., 2015). Current discussions are now investigating how niches in GI tumors contribute to the survival, growth, differentiation, migration and therapeutic resistance of CSC.

### 5.1 Immune cell moderations

TME is characterized by a chronic inflammatory state (Plaks et al., 2015). A growing body of evidence have demonstrated a negative correlation between cellular stemness and immune response within the niche (Miranda et al., 2019; Zheng et al., 2022). CSC-derived immunosuppressive factors, including various anti-apoptotic proteins, are its direct means of self-protection (Olver et al., 2006; Abdullah and Chow, 2013). Mediated by CSC-tumor system interactions, TME is exhibited as a milieu for immune detection avoidance (Clara et al., 2020). In CRC, GC and PDAC, studies have consistently highlighted a link between diminished populations of cytotoxic cells and lower survival rates, as well as unfavorable clinical outcomes (Peng et al., 2017; Yu et al., 2018; Zheng et al., 2022). Therefore, it is of great importance to understand the mechanisms orchestrating CSC-mediated immune evasion, and to elucidate the regulatory roles of immune cells in shaping CSC phenotypes.

#### 5.1.1 Tumor-infiltrating lymphocytes

Tumor-infiltrating lymphocytes, one of the major constituents of TME, refers to the lymphocytes that migrate from the blood to the site of the tumor, including CD8^+^ T cells, CD4^+^ T cells, B lymphocytes and NK cells (Yang et al., 2021). Among the trending cancer therapies, immune check point inhibitors is one of the most promising avenues. PD-L1, one of the most studied targets, can attenuate T-cell activity signals both by directly binding PD-1 and by interacting with inhibitory CD80 (Keir et al., 2008; Sun et al., 2018). Another immune checkpoint mediator, CTLA-4, competes with the CD28 receptor on T cells for binding to CD80, thereby curbing CD8^+^ T cell activity (Farhood et al., 2019).

CD8^+^ T cells are the main immune effectors exhibiting cytotoxicity. However, CSCs can escape CD8^+^ recognition by decreasing the expression of MHC-1 molecule (Morrison et al., 2018), and by elevating CD80 level in a TGFβ-dependent, CTLA-4 mediated manner (Miao et al., 2019), thereby acquiring immune resistance. Meanwhile, T cell activity is suppressed via upregulated PD-L1 expression on CSC surfaces (Wu et al., 2017b), as observed in various cancer types including CRC (Wei et al., 2019; Zheng et al., 2022) and GC (Sun et al., 2020).

In contrast, CD4^+^ T cells act not as effectors but regulators of immune responses, mediating the proliferations and activities of cytotoxic cells. Regulatory T (Treg) cells, among the CD4^+^ T cell subtypes, primarily suppress immune responses, inhibiting the cytotoxic functions of CD8^+^ T cells and NK cells (Liu et al., 2019). Particularly, the CD4^+^CD25^+^ T cells, marked by upregulated FOXP3 mRNA expression and intracellular CTLA-4 molecules, are related to poorer prognosis and survival rates in patients with GC and EC (Kono et al., 2006). The crosstalk between CSC and Treg significantly contributes to the immunosuppressive milieu of the TME. Liver CSCs, for instance, elevate Treg infiltration by upregulating PD-L1 and TGFβ1 (Wu et al., 2023). Reciprocally, Th17, another CD4+T cell subtype with occasional anti-inflammatory phenotype (Sharma et al., 2009), is found in GC, CRC and PDAC as modulator of CSC self-renewal capacity (Su et al., 2014). By releasing IL17, Th17 can induce EMT-associated mitogen-activated protein kinase (MAPK) and AKT kinase activation, further augmenting CSC properties (Becerril-Rico et al., 2021).

#### 5.1.2 Tumor-associated macrophages

Macrophages display remarkable plasticity and abundance in the CSC niche. TAMs exist with a wide range of spatial and temporal variance (Huang et al., 2019b), with TME-resided substances mediating their differentiation, dedifferentiation, and even re-education (Bowman et al., 2016). TAMs are generally defined into two polarized types, the classically activated M1 with anti-tumor properties, and the alternatively activated M2 with immunosuppressive effects (Najafi et al., 2019), the later mainly manifested in established tumors.

CSCs play a critical role in recruiting M2 macrophages. In a comprehensive comparison of 26 CSC subsets within a CRC patient cohort, M2 macrophages stood out as one of the most substantial cell types in groups exhibiting a high stemness signature (Zheng et al., 2022), indicating its close association to CSC stemness. Many pro-tumorigenic macrophage factors are elevated in a CSC-enriched environment, including CCL2 and macrophage colony-stimulating factor 1 (CSF1), the main governors of TAM survival and metastasis. In EC colony, the CCL2-CCR2 axis had been demonstrated as a critical M2 polarization factor, the blockade of which leads to decreased PD-L2 expression in TAMs and suppressed tumor invasiveness (Yang et al., 2020). Research on CRC assays underscore the similar M2-polarizing effects of CSF1, where an upregulated level of IL-10 and CCL2 was also observed, further linking CSC-derived substances with M2 population dynamics (Huang et al., 2021).

Reciprocally, TAM activity increases CSC survival rate and reinforces CSC phenotypes, as a study using HCC mouse model documented an increase in CSC-like traits in TAM coculture group (Fan et al., 2014). M2-dominated TAMs regulate immune responses within the tumor niche, actively contributing to cancer cell immune evasion. Interactions between TAM and other immunocytes display an overall immunosuppressive effect, suggesting TAM’s role in neutralizing anti-tumor properties. In GC, TAMs impair NK-cell function and compromises its cytotoxicity by downregulating the expression level of IFNγ, TNFα, and Ki-67 (Peng et al., 2017). Literature investigating TAM also suggested its close relationship with EMT. In GC, TAMs expressing CD68 correlate with reduced epithelial marker E-cadherin (Lin et al., 2015a) and upregulated forkhead box Q1 (FOXQ1) expression (Guo et al., 2017). TAM can also induce the secretion of multiple soluble factors including IL-6 and TGFβ, respectively promoting CSC self-renewal and inducing EMT (Wan et al., 2014). Interestingly, although M2 macrophages exhibit comparably more phagocytotic phenotype (Najafi et al., 2019), it does not affect CSC survival. This is mediated by the upregulation of CD47 in CSCs, with a “do not eat me” signal observed in various cancer types including HCC and PDAC (Lee et al., 2014; Cioffi et al., 2015).

#### 5.1.3 Dendritic cells

DC is the most powerful antigen presenting cell in human immune system. Mature DCs can effectively stimulate T cell toxicity and cross-present tumor antigens (Bayik and Lathia, 2021). However, DCs residing in TME often exhibit compromised maturation and display tolerogenic phenotypes. Meanwhile, DCs have been shown to promote early metastasis by inducing the proliferation of Treg and suppressing the activities of CD8^+^ T cells. In GC, DC is able to convert effector T cells into CD4^+^CD25+FOXP3+Treg (Liu et al., 2019), thus amplifying the immunosuppressive milieu. In PDAC models, DC-expressed PD-L2 and MGL2 are also found to govern tumorigenesis, the blockade of which in turn suppressed metastasis formation (Kenkel et al., 2017).

Latest evidence suggests reciprocal interactions between DCs and CSCs in GI tumors. DCs obtained from CRC patients were recorded to exhibit elevated expression level of CXCL-1, a substance responsible for activating proteins within the CSC signaling network including SOX2 (Hsu et al., 2018). On the other hand, while the precise mechanism through which CSCs enhance DC tumorigenicity remains unclear, several studies managed to establish positive connections indicating CSC’s promotive effects in tumor-associated DCs(Grange et al., 2015).

### 5.2 Cancer-associated fibroblast

**FIGURE 2:**
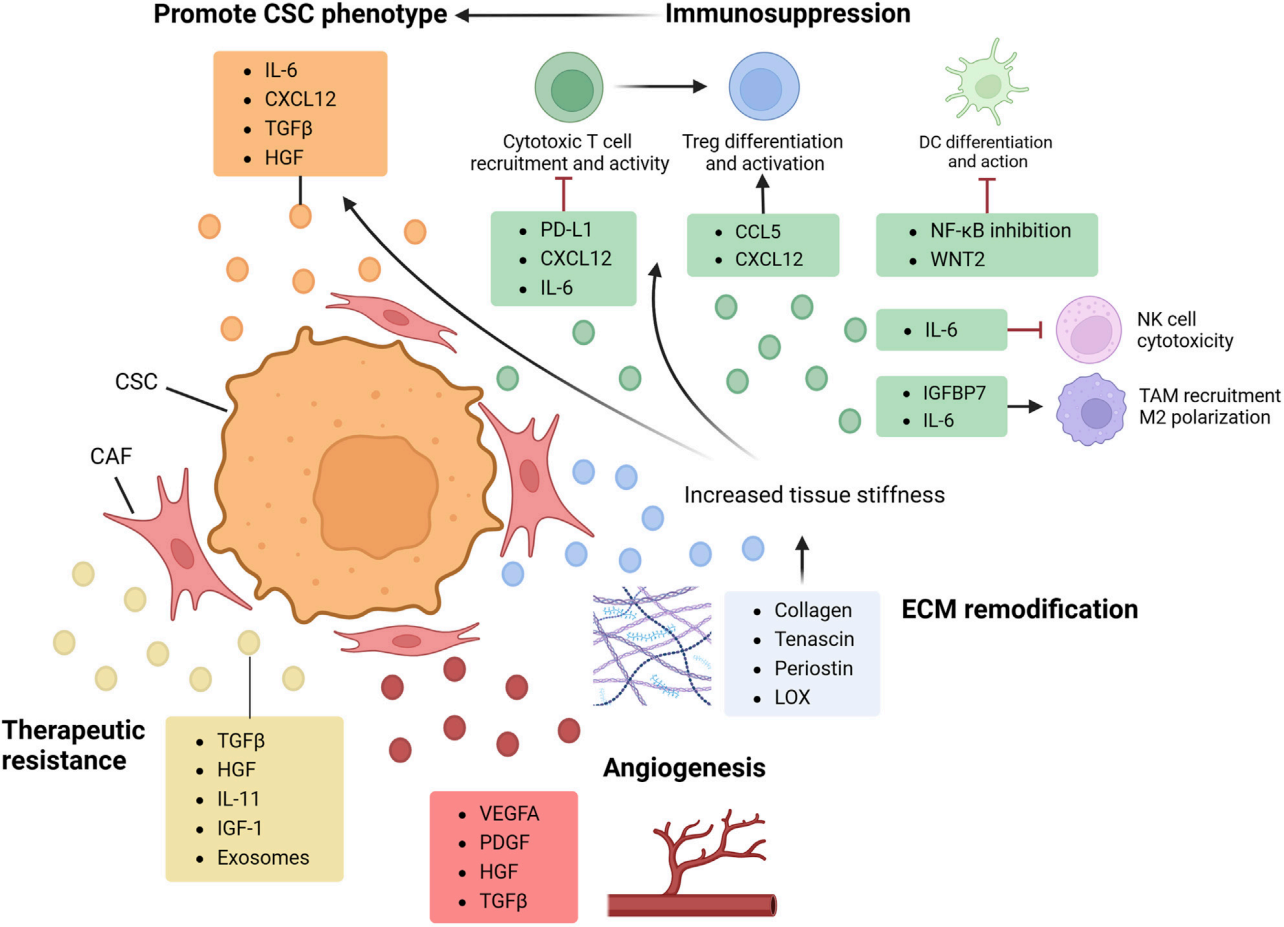
CAF in CSC regulation. By secreting multiple factors and engaging in cell crosstalk, cancer-associated fibroblast (CAFs) shapes the tumor microenvironment (TME) and orchestrate cancer stem cell (CSC) properties. CAF directly promote CSC phenotypes by producing cytokines such as TGFβ and IL-6, activating protumoral axis and upregulating CSC proliferation and clonogenicity. By interacting with immunocyte, CAF also plays a crucial role in mediating CSC immune evasion and shaping a pro-CSC immune landscape. With cytokines including IL-6, CXCL12 and CCL5, CAF can inhibit cytotoxic cells and induce pro-tumoral polarization and differentiation of macrophage and Treg (regulatory T cell). As the major producer of ECM constituents and cross-linking enzymes, CAF can remodel ECM architecture, increasing tissue stiffness and elevating CSC properties by both directly regulating its dormancy state and activating EMT, and indirectly inducing hypoxia environment and impeding immunocyte infiltration. CAF can also produce various factors promoting angiogenesis and therapeutic resistance. *CCL, CC-chemokine ligand; IGFBP, insulin-like growth factor binding protein; LOX, lysyl oxidase; PDGF, platelet-derived growth factor.*

CAFs are defined as a group of elongated cells located in cancer tissues that do not fall under epithelial, endothelial, or immune cell categories (Sahai et al., 2020). As the major constituent of ECM in GI organs (Kobayashi et al., 2019), they are characterized by high resilience (Kalluri, 2016) and heterogeneity, and are active participants in cell signaling mechanisms (Ohlund et al., 2014). CAFs have been shown to reshape ECM and establish communication networks with both tumorigenic and immune cells through the secretion of diverse chemokines and cytokines (Nurmik et al., 2020). This multifaceted interaction facilitates cell proliferation, migration, and angiogenesis, exerting a significant influence on the modulation of tumor immune responses while enhancing tumorigenic traits (Figure 1 and Figure 2).

#### 5.2.1 Promoting CSC phenotype

CAFs actively participate in CSC phenotype promotion and stemness maintenance by secreting an array of cytokines, chemokines and growth factors, including IL-6, CXCL12, TGFβ and hepatocyte growth factor (HGF).

As a key tumorigenesis factor, TGFβ stands out as a major mediator of the crosstalk between CAF and other tumor cells (Wu et al., 2013). Once activated, TGFβ pathway not only triggers fibrosis within the TME but also serves as an initiator for metastasis, reshaping the TME dynamics, fueling CSC proliferation, and propelling tumor advancement (Calon et al., 2012; Shi et al., 2020). Moreover, CAF-derived CXCL12 can bind to cancer cell-derived CXCR4, activating the CXCR4/CXCL12 axis and promoting CSC phenotypes, including its proliferation, invasion and metastasis (Orimo and Weinberg, 2006). Research targeting CRC cohorts revealed a poor prognosis related to an elevated CXCR4 and CXCL12 expression level (Saigusa et al., 2010), emphasizing their potential as malignancy markers. Studies in EC and GC have also demonstrated the cancer promoting properties of CAF-secreted IL-6, which promotes EMT and metastasis via JAK2/STAT3 signaling pathway (Wu et al., 2017a). Furthermore, various literature has reported HGF’s critical role in tumorigenesis. In HCC model, CAF can produce HGF, which regulates liver CSCs via activation of downstream effector FRA1, and promotes tumor growth process in a fibrosis-dependent manner (Lau et al., 2016). Similarly in CRC, HGF orchestrates CSC stemness via augmenting WNT pathway, restoring its phenotype even in well-differentiated tissues (Vermeulen et al., 2010).

#### 5.2.2 ECM remodification

As the main producer of collagen, tenascin and periostin (Calon et al., 2012), CAF is a key factor in the remodeling and reorganization of ECM. By secreting matrix-crosslinking enzymes, CAF can induce force-mediated reconstruction and amplify the stiffness of tumor tissues. This altered ECM architecture triggers pro-survival signaling (Rice et al., 2017) and further intensifies the proliferation, metastasis and therapy resistance of cancer cells (Schrader et al., 2011; Mohammadi and Sahai, 2018). In HCC, fibrotic liver tissues display higher proliferative indices and reduced cancer cell apoptosis, suggesting an association between increased tissue stiffness and elevated cancer cell proliferation rates. Interestingly, cell clonogenicity have been found to increase in a soft, rather than stiff, microenvironment. Schrader et al. discovered that, in HCC cells treated with chemotherapeutic agent cisplatin and 5-Fluorouracil (5-FU), an enrichment of cells with surface marker CD133, CXCR4 and CD44 is observed in the soft supports instead of the hard ones. However, this phenomenon was absent in untreated cell groups, indicating the role of CSCs specifically in response to treatment. Meanwhile, escalated tissue stiffness may trigger the collapse of vasculatures (Bordeleau et al., 2017), inducing tissue hypoxia and blocking T cell migration toward tumor sites (Salmon et al., 2012).

CAF-mediated matrix remodification is also a potent trigger of local metastasis. In a study discussing CRC metastasis, Calon et al. proposed a Darwinian model in which cells with tumor-initiating competence need to acquire TGFβ-associated functions and interact with CAF. The microenvironment shaped by TGFβ, known as the TGFβ niche, is filled with colonization-required stroma and pro-metastasis chemokines, both secreted by TGFβ-stimulated CAFs (Calon et al., 2012). Another study identified PDAC-derived SPOCK-1 as a key stromal protein, and documented its capacity to elevate cancer cell growth through matrix reorganization (Veenstra et al., 2017).

#### 5.2.3 Immunosuppression

Considering the CSC-promoting effects of immunocytes in TME, it is imperative to investigate CAF’s engagement in immunocyte crosstalk as an indirect means of CSC regulation, including its role in evading immune surveillance and shaping the tumor immune landscape.

By interacting with collagen and reorganizing ECM structure, CAF can impede immune cell immigration (Sahai et al., 2020). Abnormal vasculature blocks T cell extravasation, while an altered intercellular adhesion state may disrupt functional diapedesis (Turley et al., 2015). CAF also modulates the immune landscape via the production of PD-L1, TGFβ, CCL5 and CXCL12 (Ng et al., 2005; Ahamed et al., 2008; Turley et al., 2015). These cytokines collectively contribute to shaping an immunosuppressive TME, either by affecting downstream pathways, recruiting immunosuppressive factors, or by directly regulating immune cell properties.

CD8^+^ cytotoxic T cell activity is inhibited by CAF-derived TGFβ, CXCL12 and IL-6. While CAF can directly produce PD-L1 (Turley et al., 2015), substances like TGFβ and CXCL12 can also attenuate the anti-PD-L1 response (Cheng et al., 2018; Mariathasan et al., 2018). Moreover, CAFs recruit and activate FOXP3+ Tregs to suppress antitumor immune responses (Wang et al., 2023). CAF-derived CCL5 is found to promote the survival, differentiation and activation of Tregs (Wang et al., 2017; Kieffer et al., 2020), further restraining immune surveillance. In HCC and PDAC, CAFs induce M2 polarization of macrophages, fostering an immunosuppressive environment (Mathew et al., 2016; Chen et al., 2021). By upregulating the level of IL-10, CAF in PDAC is found to activate Arg1 expression in macrophages, hence inducing alternative polarization. Furthermore, CAF in GC has been shown to secrete insulin-like growth factor binding protein 7 (IGFBP7), elevating TAM infiltration level and promoting tumor growth (Li et al., 2023a). Notably, TAMs are engaged in a crosstalk with CAFs, producing factors that further activate CAFs (Comito et al., 2014) and creating an intercellular network that fuels cancer growth and metastasis (Sathe et al., 2023).

#### 5.2.4 Angiogenesis

Neovascularization, one of the major conditions for CSC maintenance, is regulated by various CAF-derived factors. As a critical angiogenetic factor, vascular endothelial growth factor A (VEGFA) is directly produced by CAFs (Kobayashi et al., 2019) or indirectly activated by CAF-secreted molecules (Wang et al., 2019a). A study in CRC discovered that, combined blockade of VEGFR and COX2 augmented antiangiogenic, antitumoral and anti-CSC effects, indicating VEGF’s role in maintaining CSC properties (Zhao et al., 2015a). Other pro-angiogenic substances secreted by CAFs include platelet-derived growth factor (PDGF), CXCL12, fibroblast growth factor 2 (FGF2), HGF and TGFβ (Anderberg et al., 2009; Wang et al., 2019a; Kobayashi et al., 2019; Hjazi et al., 2023).

#### 5.2.5 Therapeutic resistance

An extensive body of research underscores the pivotal contribution of CAFs in fostering therapeutic resistance in cancer cells. Remodeled by CAF, the dense ECM acts as a physical barrier to impede drug deliveries. A study using PDAC mice model revealed notably elevated interstitial fluid pressures (IFP) and occurrences of vascular collapse in tumor tissues, (Provenzano et al., 2012), which are presented as delivery barriers induced by CAF’s desmoplastic reactions.

Various CAF-secreted soluble factors also play significant roles in eliciting therapeutic resistance of GI tumors (listed in Table.1). These factors exert their effects by either obstructing therapy pathways, inducing rapid adaptations to altered milieu, or accelerating damage repair processes. Meanwhile, CAF’s immunosuppressive properties, highlighted earlier, dampen the responses to immune checkpoint inhibitors, consequently diminishing the efficacy of conventional immunotherapies.

**TABLE 1:** CAF-secreted soluble factors in therapeutic resistance of GI tumors.

Secreted factor	Targeted pathway	Mechanism	Tumor type	Citation
IGF-1	IGF1/IGF1R	Induces metabolic changes; reduces cancer cell sensitivity to irradiation	CRC	Tommelein et al. (2018)
CXCL1	Mek/Erk	Regulates an ROS-dependent DNA damage repair	EC	Zhang et al. (2017b)
TGFβ	FOXO1/TGFβ1	Elevates epithelial cell proliferation and angiogenesis	EC	Zhang et al. (2017a)
HGF	ERK–MAPK	Provides BRAF-independent mechanism regarding BRAF-targeted therapies; resist tyrosine kinase inhibitors	CRC	Straussman et al. (2012), Luraghi et al. (2014)
IL-11	JAK/STAT3/Bcl2	Activates anti-apoptosis signaling pathway	GC	Ma et al. (2019a)
IL-17A	-	Induces viability of CIC	CRC	Lotti et al. (2013)
lncRNA H19	β-catenin	Competes with stemness-inhibiting factor miR-141	CRC	Ren et al. (2018)
miR-522	ALOX15	Suppresses ferroptosis	GC	Zhang et al. (2020)
Proteins (unidentified)	mTOR/4E-BP1	Secretes proteins that activate tumor cell survival	PDAC	Duluc et al. (2015)
Exosomes (unidentified)	WNT	Increases the percentage, clonogenicity and growth of CSCs	CRC	Hu et al. (2015)

### 5.3 Angiogenesis and endothelial transformation

In TME, stress-induced angiogenesis provides a conducive niche for CSCs, while endothelial cells themselves exhibit properties that foster stemness (Yang et al., 2021). Studies on early tumor stages highlighted the spatial proximity between CSC niche and endothelial cells (Mercurio, 2019), indicating a reciprocal relationship established by paracrine signaling.

VEGF, a potent endothelial cell-specific mitogen, orchestrates endothelial development and pathological angiogenesis (Leung et al., 1989). It also plays the role of CSC promoter, as VEGF blockade results in declined CSC population and activities (Mercurio, 2019). Previous research primarily focused on squamous carcinoma and skin cancer, while recent findings in GC mouse models uncovered a KRAS-activated VEGF production in CSC (Yoon et al., 2023), suggesting CSC’s role in regulating endothelial growth and neovascularization. Thus, anti-vascular therapies emerge as a promising approach to combat cancer metastasis (Lu et al., 2013).

Meanwhile, endothelial cells can perform CSC-promoting properties. Previous study has identified that Jagged-1, a soluble factor secreted by CRC endothelial cells, activates the Notch signaling pathway and promote CD133+ CSC self-renewal and proliferation *in vitro* and *in vivo* (Lu et al., 2013). Moreover, recent literature correlates a reduction in CSCs to the inhibition of a CD44 ligand, DLL4, derived from endothelial cells. Meconca et al. demonstrated that suppressing DLL4 in endothelial cells, even during induced tumoral neovascularization, resulted in insubstantially perfused vessels unable to support normal vascular functions, thereby inhibiting EMT and CSC activity (Mendonça et al., 2019).

### 5.4 Hypoxia

Aberrant ECM structure and vasculature in tumor tissues frequently leads to hypoxia, a major stressor that drives the adaptive properties and tumorigenicity of CSCs. Consequently, hypoxic environment favors cells with CSC-like features due to clonal selection, fostering malignant properties (Kim et al., 2018). A study using differential gene expression analysis revealed a strong correlation between tumor diversity and hypoxia-related genes (Ma et al., 2019b). Upregulated levels of hypoxia-inducible factor-1α (HIF1-α), when stabilized by CAF-derived TGFβ, can activate the expression of SHH transcription factor GLI2 in colorectal CSCs, consequently maintaining CSC stemness, promoting dedifferentiation and metastasis (Tang et al., 2018). In various cancers, other proposed mechanisms including the ROS-activated stress response pathway (Liu et al., 2008) and the Notch signaling pathway (Quail et al., 2012) further associate hypoxia with the increased stemness, survival rate and functions of CSC.

The hypoxic environment directly increases HIF1-α levels, triggering downstream hypoxia signaling and stimulating the production of VEGF (Goel and Mercurio, 2013), which plays a major role in angiogenesis and TAM recruitment (Kitamura et al., 2015). Infiltrating TAMs, once migrated to the site, engage in crosstalk with VEGF, further enhancing VEGF-A production and promoting tumor angiogenesis. Additionally, hypoxic CSCs impede T cell activities by producing STAT3, which acts as a transcriptional source for HIF1-α and VEGF, thus exhibiting a hypoxia-induced immunosuppressive effect (Wei et al., 2011).

## 6 Therapies targeting stemness regulation

Conventional cancer therapies, including chemotherapies and radio therapy, are all currently challenged with severe limitations due to the failure to eliminate CSCs. Without proper eradication, dormant CSCs can be reactivated to trigger cancer relapse. Therefore, novel therapeutic researches are focusing on inhibiting CSCs by targeting the substances engaged in CSC stemness regulation.

### 6.1 Immunotherapy

#### 6.1.1 Antibodies targeting biomarkers

Bispecific antibodies (BiAbs), representing an innovative strategy in immunotherapy, can simultaneously bind both T cell and tumor cells expressing specific antigens. The binding activates an MHC-independent immune response leading to cell lysis, thereby exhibiting antitumor effects regardless of tumor cell’s immune evasiveness (Bargou et al., 2008). Similar approach can be employed to target CSCs utilizing CSC-specific surface markers such as CD133 and EpCAM.

Targeting CD133, Zhao et al. devised a CD133 monoclonal antibody (MS133) with dual-antigen-binding specificity to CD133 and CD3. MS133 exhibited significant cytotoxicity in CD133^high^ CRC cells, demonstrating enhanced stability and prolonged cytotoxic activity (Zhao et al., 2015b). Furthermore, solitomab, a single chain antibody, can bispecifically target EpCAM and CD3. Preclinical evidence revealed its potential in reducing EpCAM + cell group in PDAC and EC both *in vitro* and *in vivo* (Cioffi et al., 2012; Yu et al., 2021). Clinically, a Phase Ⅰ study in patients with refractory solid tumors (including CRC and GC) reported a modest inhibitory effect of solitomab on the metastasis and progression of EpCAM + cancer (Kebenko et al., 2018). Similarly, catumaxomab, a drug originally approved for treating malignant ascites, targets on EpCAM and CD3 in late-stage GI tumors. Though withdrawn from the market in 2017, associated studies on catumaxomab have not stopped, with ongoing clinical trials focusing on its efficacy in GC patients (NCT04222114, NCT01504256).

#### 6.1.2 CSC-based vaccines

Compromising DCs’ antigen presentation function is one of the most important strategies in CSC immune evasion. However, CSC-based vaccines can directly expose the CSC-unique antigens to the immune system, enhancing CSC recognition and activating robust immune effects (Hashemi et al., 2023), regardless of the decreased MHC-1 expression. The induction of strong immune responses can also elicit an immune memory, which reduces the likelihood of cancer recurrence (Kishi et al., 2021; Hashemi et al., 2023). The therapy features the injection of processed CSCs from autologous or allogeneic origins. The injected substance could be whole CSC-based vaccines made from previously inactivated CSCs, or CSC-lysates loaded in antigen-presenting cells (APCs), mostly DCs (Izadpanah et al., 2023).

The efficacy of this novel approach is supported by abundant preclinical evidence (Perez-Banos et al., 2023). In CRC mouse models, a whole-CSC vaccine made from CD133+ CSC lysates triggered the increase of NK cell cytotoxicity and secretion of inflammatory chemokines such as IFN-γ, which subsequently inhibits the proliferation of CD133+ and ALDH + cell groups, indicating a reduction in CSC proliferation (Guo et al., 2020). In HCC mouse models, Li et al. demonstrated that DC vaccine induced T cell activity *in vivo*, resulting in decreased tumor mass growth (Li et al., 2016). Enhanced T cell cytotoxic activity was observed in GC cell lines when utilizing a DC-CSC mRNA vaccine in cell co-culture (Bagheri et al., 2019).

Progress in clinical studies also supported the potential of CSC-based vaccines. In the first clinical trial of pancreatic CSC vaccine launched by Lin et al., a pancreatic CSC-derived vaccine was injected at low, medium and high dose to three groups of PDAC patients. With a significantly obvious response in the high-dose group, this trial preliminarily validates the efficacy of CSC vaccine as a PDAC therapy (Lin et al., 2015b). Moreover, Lau et al. carried a Phase Ⅰ study featuring patients with PDAC resection. The injected vaccine, combining autologous DC and allogeneic CSC lysates, has shown a promising outcome in containing cancer metastasis rate (Lau et al., 2022). Ongoing clinical trials are also investigating the efficacy of CSC vaccines in other GI malignancies, including CRC, GC and HCC (NCT01885702, NCT04912765, NCT04147078). Further researches are required regarding DC subtypes and their interactions with substances in the TME, and a refined categorization of CSC biomarkers is needed (Izadpanah et al., 2023) to ensure the targeting efficacy and precision of the vaccines.

#### 6.1.3 CAR therapy

The chimeric antigen receptor (CAR) therapy features the genetic modification of cytotoxic cells, mostly T cell or NK cell. Instead of targeting MHC in the conventional way, the modifiable extracellular region serves as a chimeric receptor, which enables the cell to bind any surface antigen of interest, making it a customizable tool to directly target tumor associated markers (Garza Trevino et al., 2023). This flexibility makes CAR therapy a versatile tool in detecting and eliminating CSCs, with CD133 and EpCAM as the most recruited CSC markers to date.

Preclinical studies highlight the efficacy of CAR therapy in anti-tumor activities, especially in combination with chemotherapy. Han et al. tested the efficacy of anti-CD133 CAR-T and cisplatin combination therapy in GC xenograft models (Han et al., 2021). Cisplatin is a first line chemotherapeutic drug against GC, but has shown limited effect due to CSC stemness and drug resistance, even upregulating CD133 expression when applied alone. However, with anti-CD133 CAR-T, cisplatin effectively induces the release of cytokine, and with the pronounced cytotoxicity of CAR-T cells, they jointly diminish the growth and infiltration of CD133^+^ cell. Moreover, Zhang et al. investigated the combination therapy of regorafenib and CAR-NK-92 cells in established CRC models (Zhang et al., 2018). Targeting EpCAM^+^ cells significantly increased cytokine levels and suppressed tumor growth, compared to monotherapy with regorafenib or CAR-NK-92.

In a Phase Ⅰ study of CAR-T cell directed against CD133 (CART-133), patients with advanced solid tumor (PDAC, CRC, HCC) were enrolled and infused with CART-133. Tissue biopsy revealed effective elimination of CD133 cell population, and the trial concluded promising disease stable rate and progression-free survival of CART-133 (Wang et al., 2018b). Another CART-133 Phase Ⅱ study conducted by Dai et al. has reached similar result. The trial recruited advanced HCC patients, and also recorded promising clinical response and manageable CART-133 safety profile. The level of prognostic indicators, such as proangiogenic and inflammatory factors, were also tracked and reported (Dai et al., 2020).

Further investigation and application of CAR therapy requires comprehensive research on CSC biomarkers, as a large percentage of CSC markers are also expressed on normal tissue cells (Kim and Ryu, 2017). Therefore, recognition of CSC-specific antigens needs to be improved regarding tissue-specificity to enhance therapy efficacy and to avoid on-target/off-tumor toxicity (Izadpanah et al., 2023).

#### 6.1.4 Immune check point inhibitor

As previously highlighted, the overexpression of immune check points, notably CTLA-4 and PD-L1, is crucial in CSC’s immune evasion. Thus, inhibiting the immune check points could be an effective approach to reinvigorate anti-tumor immune responses.

Multiple ongoing clinical trials are investigating the safety and feasibility of existing drugs, as well as potential combined therapies. Nivolumab is a PD-1 inhibitor that shows high clinical overall survival rate, and has been applied as first-line solid tumor chemotherapy. It is frequently used in combination therapy with ipilimumab, a human mono-antibody that blocks CTLA-4 activation. A randomized Phase Ⅲ study showed that, in untreated patients with EC and GC, the nivolumab and ipilimumab combined therapy achieved a significant improvement in overall survival rate (Janjigian et al., 2021). Similarly, a Phase II study in CRC patients exhibited promising progression-free outcomes with the nivolumab plus ipilimumab combination (Lenz et al., 2022). A randomized trial investigating HCC resection’s postoperative use of this therapy also concluded a lower recurrence rate, further expanding its application scope (Kaseb et al., 2022). The effect of other immune check point inhibitors in GI cancer, including CXCR4 inhibitor plerixafor, PD-1 inhibitor camrelizumab and pembrolizumab, are all investigated by ongoing clinical trials (Muro et al., 2016; Doi et al., 2018; Xu et al., 2019; Parikh et al., 2021; Li et al., 2023b) (NCT02179970, NCT02658019).

### 6.2 Targeting signaling pathways

CSCs rely on multiple cellular signaling pathways to maintain its stemness and tumorigenicity. Therefore, therapies targeting aberrantly triggered pathways can downregulate these tumoral effects and eliminate CSCs for cancer eradication.

#### 6.2.1 Salinomycin

Salinomycin, a natural polyether product isolated from *Streptomyces albus*, has been suggested by many preclinical researches as a potent anti-tumor drug. Salinomycin effectively blocks WNT pathway by suppressing the formation of the β-catenin/TCF4E complex, subsequently downregulating the expression of WNT-associated genes LEF1 and LGR5 (Wang et al., 2019c) and inhibiting the growth of cancer cells. Moreover, salinomycin is known as an inhibitor of ATP-binding cassette (ABC) transporters, thereby compromising the drug resistance displayed by CSCs. It also exerts direct effects on CSCs by inducing cell apoptosis and DNA damage, or by promoting CSC differentiation (Naujokat and Steinhart, 2012).

Preclinical studies have highlighted salinomycin as a promising agent for eliminating CSC in CRC, GC and HCC (Dong et al., 2011; Zhi et al., 2011; Wang et al., 2020c). Additionally, combination therapies have gained interests in experimental studies. In PDAC models, Zhang et al. demonstrated that simultaneous administration of salinomycin and gemcitabine, a first-line drug commonly targeting non-CSC tumor cells, resulted in better containment of tumor growth and cancer cell viability compared to using either drug alone (Zhang et al., 2011). Current studies are also exploring novel drug delivery methods including nanocapsules or nanocrystals, with evidence validating for their capacity to enhance efficacy and bioavailability in CRC patients (Wang et al., 2020b; Tsakiris et al., 2020).

#### 6.2.2 Sulforaphane

Sulforaphane is an isothiocyanate found in cruciferous vegetables like broccoli and cauliflower. Extracted and purified sulforaphane is reported to exert cancer-preventive functions including apoptosis induction, angiogenesis inhibition and metastasis suppression (Elkashty and Tran, 2021). Recent evidence suggests sulforaphane as a potent CSC inhibitor across multiple cancer types, as it targets crucial signaling pathways including WNT, Notch, SHH and NF-κB (Naujokat and McKee, 2021). Moreover, in PDAC, sulforaphane is found to deter CSC self-renewal by inhibiting EMT-associated proteins such as vimentin, TWIST-1 and ZEB1 (Srivastava et al., 2011).

Sulforaphane administration in PDAC xenograft models was associated to NF-κB binding and subsequent apoptosis activation. In the preclinical study conducted by Kallifatidis et al., treated groups are found with suppressed angiogenesis and tumor growth, with no apparent cytotoxicity observed in normal cells (Kallifatidis et al., 2009). Tracking SHH pathway components *in vivo* also reveals sulforaphane’s function in SHH blockade, accompanied with reduced PDAC tumor size and inhibited EMT activities (Li et al., 2013). Sulforaphane’s anti-tumor effects has also been preclinically demonstrated in CRC and HCC (Liu et al., 2017; Ren et al., 2017; Chen et al., 2018; Chen et al., 2020). Large-scale clinical transformation, however, is challenged by the limited efficiency of drug delivery (Elkashty and Tran, 2021). The most recent clinical investigation is conducted by Lozanovski et al., with capsules of pulverized broccoli sprouts (containing 90 mg/508 μmol sulforaphane) administered to PDAC patients (Lozanovski et al., 2020). Though the results were not statistically significant, treatment group showed slightly prolonged survival, which provides preliminary evidence for the feasibility of sulforaphane application against GI cancer.

#### 6.2.3 Napabucasin

Napabucasin, also known as BBI608, is an orally available STAT3 inhibitor. By reducing STAT3-induced gene transcription, it can exert anti-CSC effects and effectively prevent cancer metastasis and recurrence (Sonbol et al., 2019). Li et al. demonstrated its capability to impede spherogenesis and eliminate CSC-like cells, as well as inhibit cancer metastasis and relapse (Li et al., 2015b). These effects are observed across multiple cancer cell lines, including those originated from liver, pancreas and colon, suggesting napabucasin as a potent CSC-inhibitor in GI malignancies. Further evidence indicates that the administration of napabucasin facilitates the overall responsiveness of cancer cells to conventional therapies (Flebbe et al., 2022), providing new strategies for designing efficient combination therapies.

In a Phase Ⅰb/Ⅱ study, the combination therapy of napabucasin and bevacizumab was administered to patients with metastatic CRC, which demonstrated prolonged survival rate and contained tumor growth in treated group compared to placebo group (Bendell et al., 2017). In an international multi-center randomized Phase Ⅲ study (NCT02753127) with 1253 CRC patients, napabucasin efficacy in combination with 5-FU, Leucovorin, and Irinotecan (FOLFIRI) was evaluated among adult patients (Grothey et al., 2016). Another open label, multi-center, Phase Ⅰ/Ⅱ dose escalation study (NCT02024607) included 495 adult patients with advanced GI tumor. Results highlighted that napabucasin administration effectively controlled cancer metastasis and significantly promoted progression-free survival rate. However, several completed clinical trials fail to acquire anticipated outcomes, including a Phase Ⅲ study on PDAC (NCT02993731) and the Phase Ⅲ BRIGHTER trial on GC (NCT02178956). These studies either detected no significant differences in treatment groups or recorded reverse effects.

### 6.3 Targeting ncRNA

NcRNAs can regulate multiple CSC properties, including cell stemness, therapy resistance and metastatic abilities. Therefore, targeting ncRNAs, either by silencing or upregulating specific ncRNAs, stands as a promising avenue for novel therapeutic approaches (Hwang et al., 2021). In a Phase Ⅰ study in patients with advanced solid tumors, MRX34, a liposomal miR-34a mimic, was intravenously administered in a dosage escalating regimen (Hong et al., 2020). Despite participants encountering manageable adverse effects including fever, chills, and nausea, the treatment showcased a dose-dependent capability to downregulate targeted oncogenes. Though the trial was terminated due to fatal immune-mediated adverse effects, it provided preliminary proof validating the feasibility of miRNA-based therapies. Moreover, a multitude of ongoing clinical studies use ncRNAs as a prognostic tool in the early detection of GI cancers, including Project CADENCE in Singapore (NCT05633342), the Sibling AN Study in Hong Kong, China (NCT01593098), and a single blind, case control, multi-center study in Shanghai, China (NCT05431621).

## 7 Conclusion

Extensive research efforts have shed light on the profound impact CSCs exert in the initiation, progression, metastasis and recurrence of malignancies. This theory, accommodating the factors behind tumor heterogeneity, has no doubt enriched our understanding of the molecular processes underlying cancer progressions. Emerging researches targeting CSC regulation has provided new insights in recruiting prognostic tools and developing novel therapeutic targets.

However, investigations into CSCs confront multifaceted challenges. Identifying CSCs primarily relies on cell surface markers, a methodology prone to inaccuracy due to their non-universal distribution. Even widely-located markers, such as CD44 and CD133, have been found to display diverse effects influenced by spatial and temporal heterogeneity (Huang et al., 2013; Miwa et al., 2017). Thus, questions addressing a more reliable strategy for CSC identification urge answers, including the number of exhibited markers needed to signify a CSC population; whether each cancer type possesses an exclusive set of markers; and whether CSC biomarker distribution alter with cancer progression.

Moreover, current findings on CSC regulation often overlook the heterogeneity among GI cancers. Due to the plasticity of CSC and the dynamic TME, regulators could exhibit diverse effects regarding spatial or temporal differences. Certain signaling pathways exhibit both tumorigenicity and anti-tumor effects (Katsuno et al., 2012; Esebanmen and Langridge, 2017; Peng et al., 2022), so the complete blockade of which may diminish its effect in tumor suppression. Furthermore, the intricate interplay within the TME suggests that inhibiting one factor might alter the effectiveness of pathways, making therapeutic outcomes unpredictable. On the other hand, this interactive complexity could lead to ineffectiveness in targeting sole factor, as alternative pathways could compensate tumorigenesis.

Therefore, dynamic updates are needed regarding CSC heterogeneity and the comprehensive interactions among influencing factors. Meanwhile, novel cancer treatments could recruit a comprehensive strategy, combining CSC eradication, blockade of regulatory pathways and chemo-sensitization, while customizing therapies based on specific cancer types (Chen et al., 2013a; Du et al., 2022).
